# Fibroma of the Tendon Sheath Arising From the Flexor Digitorum Superficialis Tendon

**Published:** 2019-01-14

**Authors:** Mary M. Jordan, Ryan Accomazzo, Gabriel Gaweda, Richard S. Zeri, Tom Reisler

**Affiliations:** ^a^Department of Surgery, The Brody School of Medicine, East Carolina University, Greenville, NC; ^b^Division of Plastic and Reconstructive Surgery, Department of Surgery, The Brody School of Medicine, East Carolina University, Greenville, NC

**Keywords:** fibroma of the tendon sheath (FTS), giant cell tumor, neoplasm, ganglion cyst, lipoma

## DESCRIPTION

**Figure 1:**
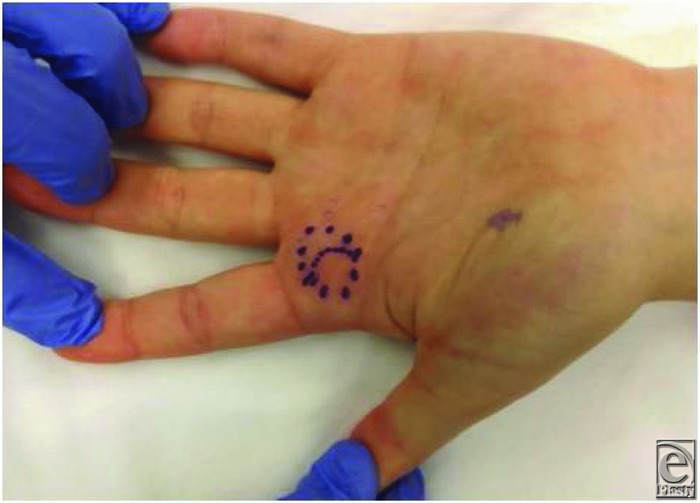
Preoperative photograph of the patient displaying the location and size of the palpable lesion, outlined at the base of the palmer aspect of the left index finger.

A 23-year-old woman presented with a 1 year of slow-growing soft tissue nodule on the volar aspect of the base of the left index finger (Fig 1). Gradually, the mass begun to limit the finger mobility and associated occasional mild localized pain. Clinical examination revealed a firm swelling with deep fixation but no skin involvement. No evidence of finger triggering was present; however, there was palpable crepitus upon pressing against flexor A1 pulley when flexing and extending the index finger.

## QUESTIONS

1. What is the differential diagnosis of a hand mass?
2. What is fibroma of the tendon sheath?
3. What is the clinical presentation of fibroma of the tendon sheath?
4. What is the appropriate treatment?

## DISCUSSION

**Figure 2:**
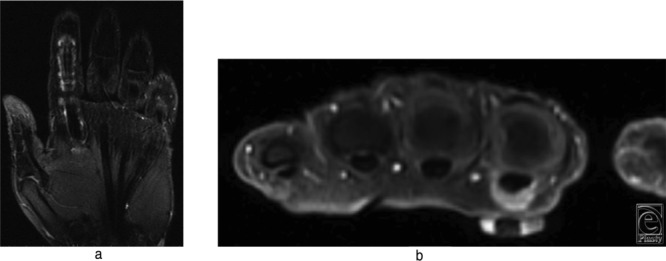
(a) Magnetic resonance scan with gadolinium-contrast coronal section of the patient demonstrating left index finger flexor tendon tenosynovitis extending from the level of the junction of the mid and distal third of the metacarpal shaft to the level of the insertion for the flexor digitorum superficialis tendon. (b) Gadolinium-enhanced thickening of the A1 pulley seen in the transverse section. Interestingly, no flexor digitorum superficialis tendon mass was visible on the magnetic resonance scan.

Certain tumors are more likely to be located in the soft tissue in the hand than in other parts of the body. All forms of soft tissue lesions must be considered in the differential diagnosis. This includes lipoma, lipofibromatous hamartoma, epidermal cyst, neuroma, angiomyolipoma, nonskeletal chondroma, synovial sarcoma, ganglion cyst, Dupuytren disease, fibromatosis, and tenosynovial giant cell tumor. The clinical features of tenosynovial giant cell tumor are particularly similar to those of fibroma of the tendon sheath (FTS). Both are likely to occur in the fingers with attachment to tendon sheath, possess similar magnetic resonance imaging (MRI) signals, and have firm, well-circumscribed, multilobulated gray-white appearance^1^^,^^2^ (Fig 2). However, FTS is distinguished from a tenosynovial giant cell tumor by its histopathologic features.

Fibroma of the tendon sheath is an uncommon, benign, slow-growing, solitary, neoplastic condition consisting of well-circumscribed, lobulated fibrocartilaginous tissue attached to tendon or tendon sheath. It typically develops in the hands, wrists, and fingers but can also occur, albeit rarely, in the lower extremities. The volar aspect of the thumb, index, and middle fingers is the commonest site of origin. FTS commonly presents within the third to fifth decades of life, with a predominance in males over females (M:F ratio of 3:1).^3^

The clinical presentation of FTS often occurs years after its formation as a painless, slowly growing mass that may irritate the surrounding tissues by compression.

About one-third of the cases present with tenderness and mild pain due to compression of the nerves underlying FTS.^4^ Fibroma of tendon sheath has also been reported to cause a “trigger wrist,”^5^^-^7 an impingement of the tumor adhering to the flexor tendons in the carpal canal, resulting in snapping finger or carpal tunnel syndrome. There have also been reports of the tumor causing complete flexion limitation of the finger.

Less than 10% of patients have reported a history of trauma.

**Figure 3:**
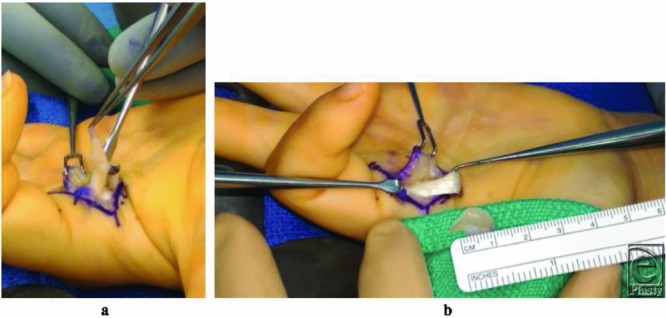
(a) Intraoperative photograph. Proximal half of the flexor A1 pulley was divided longitudinally and the FDS tendon is pulled out with a Ragnell retractor, exposing the swelling that was palpable underneath the A1 pulley. A 1-cm gelatinous, avascular, tightly adherent mass to the palmer aspect of the FDS tendon can be seen. (b) The mass was sharply shaved off the FDS tendon, incorporating a thin layer of the flexor tendon, as there was no well-defined plane between the 2 structures. FDS indicates flexor digitorum superficialis.

Treatment consists of local excision with preservation of important anatomical structures. The excision of the tumor is difficult at times because of its adherence to tendinous structures, and it is aimed to relieve symptoms but preserve function (Fig 3). Approximately 25% of these lesions have been reported to recur following surgical excision, presumably due to failure of removal of all lobules at the initial surgery. These tumors, however, do not recur aggressively, as there is no evidence of their malignant transformation or mitosis, and they do not metastasize.^8^
